# Cold Atmospheric Plasma Changes the Amino Acid Composition of Solutions and Influences the Anti-Tumor Effect on Melanoma Cells

**DOI:** 10.3390/ijms22157886

**Published:** 2021-07-23

**Authors:** Stephanie Arndt, Fadi Fadil, Katja Dettmer, Petra Unger, Marko Boskovic, Claudia Samol, Anja-Katrin Bosserhoff, Julia L. Zimmermann, Michael Gruber, Wolfram Gronwald, Sigrid Karrer

**Affiliations:** 1 Department of Dermatology, University Medical Center Regensburg, Franz-Josef-Strauss Allee 11, D-93053 Regensburg, Germany; petra.unger@ukr.de (P.U.); sigrid.karrer@ukr.de (S.K.); 2 Institute of Functional Genomics, University of Regensburg, Am BioPark 9, D-93053 Regensburg, Germany; Fadi.Fadil@ukr.de (F.F.); katja.dettmer@ukr.de (K.D.); marko.boskovic@chemie.uni-regensburg.de (M.B.); claudia1.bogner@ukr.de (C.S.); wolfram.gronwald@ukr.de (W.G.); 3 Institute of Biochemistry, University of Erlangen-Nuernberg, D-91054 Erlangen, Germany; anja.bosserhoff@fau.de; 4 Comprehensive Cancer Center Erlangen-EMN (CCC ER-EMN), D-91054 Erlangen, Germany; 5 Terraplasma GmbH, D-85748 Garching, Germany; zimmermann@terraplasma.com; 6 Department of Anesthesiology, University Medical Center Regensburg, Regensburg, Franz-Josef-Strauss Allee 11, D-93053 Regensburg, Germany; michael.gruber@ukr.de

**Keywords:** cold atmospheric plasma (CAP), plasma-treated solution (PTS), melanoma, anti-tumor, amino acid, apoptosis, senescence

## Abstract

Cold Atmospheric Plasma (CAP) is an ionized gas near room temperature. Its anti-tumor effect can be transmitted either by direct treatment or mediated by a plasma-treated solution (PTS), such as treated standard cell culture medium, which contains different amino acids, inorganic salts, vitamins and other substances. Despite extensive research, the active components in PTS and its molecular or cellular mechanisms are not yet fully understood. The purpose of this study was the measurement of the reactive species in PTS and their effect on tumor cells using different plasma modes and treatment durations. The PTS analysis yielded mode- and dose-dependent differences in the production of reactive oxygen and nitrogen species (RONS), and in the decomposition and modification of the amino acids Tyrosine (Tyr) and Tryptophan (Trp). The Trp metabolites Formylkynurenine (FKyn) and Kynurenine (Kyn) were produced in PTS with the 4 kHz (oxygen) mode, inducing apoptosis in Mel Im melanoma cells. Nitrated derivatives of Trp and Tyr were formed in the 8 kHz (nitrogen) mode, elevating the p16 mRNA expression and senescence-associated ß-Galactosidase staining. In conclusion, the plasma mode has a strong impact on the composition of the active components in PTS and affects its anti-tumor mechanism. These findings are of decisive importance for the development of plasma devices and the effectiveness of tumor treatment.

## 1. Introduction

Cold Atmospheric Plasma (CAP) is of interest in a variety of medical applications, such as sterilization [1], wound healing [2,3], virus inactivation [4] and cancer treatment [5]. The mechanisms of plasma–cell interactions are not yet fully understood, but reactive oxygen species (ROS) and reactive nitrogen species (RNS) are well known to mediate the effects of CAP treatment on cells [6,7]. The composition of reactive oxygen and nitrogen species (RONS) depends on the plasma source used, the operation parameters and the gas composition applied. Different studies have proven the safety of CAP application with the use of genotoxicity and mutagenicity assays [8,9,10,11,12]. On the other hand, various in vitro and animal studies have shown efficacious anti-proliferative and pro-apoptotic effects on tumor cells after CAP treatment [13,14,15,16,17,18]. To date, no medical plasma device has been approved for the treatment of cancer. The clinical experience gained so far is mostly based on observations of palliative treatment, and is mainly related to morphological effects on the surface of tumors of the head and neck [19,20,21]. In addition to the direct CAP treatment of tumor cells or tumor tissues, the use of plasma-treated solutions (PTS) has been more and more successful in treating tumor cells [22,23,24,25,26], despite their weaker anti-tumor effects than those of direct cell treatment [27]. However, indirect plasma treatment may have the advantage of targeting cancer cells inside the body because of its injection into the tumor tissue. The typical solutions used for the generation of PTS are cell culture media containing, among others, substantial amounts of free amino acids. The relatively short-lived RONS produced in the medium after CAP treatment may be converted into other relatively long-lived species, such as nitrite (NO_2_^−^) and nitrate (NO_3_^−^) or hydrogen peroxide (H_2_O_2_). H_2_O_2_, NO_2_^−^ and NO_3_^−^ have been described as the three main active components responsible for the anti-tumor effects of PTS [28], as other short-lived species are quenched very rapidly [29]. However, an H_2_O_2_ solution alone does not result in the same anti-tumor effect as seen after CAP treatment [30,31], suggesting that unidentified components in CAP-treated solutions support this anti-tumor effect. Conventional cell culture media such as Dulbecco’s modified Eagle’s medium (DMEM) contain different amino acids, inorganic salts, vitamins, and other substances. Consequently, it is necessary to study the interaction between CAP and these components to understand the mechanism underlying the anti-tumor effect of PTS.

In this study, we focused on the amino acids in PTS because of the already known changes in their composition after CAP treatment [32,33,34]. However, a connection between the observed changes in the amino acids and their effect on the behavior of tumor cells has not yet been investigated in detail.

## 2. Results

The aim of the study was to examine the molecular and cellular reactions of tumor cells induced by PTS that had been generated by means of the plasma care^®^ device using frequencies of 4 kHz (oxygen mode) and 8 kHz (nitrogen mode).

### 2.1. Reactive Species in DMEM + FBS PTS

**Figure 1:**
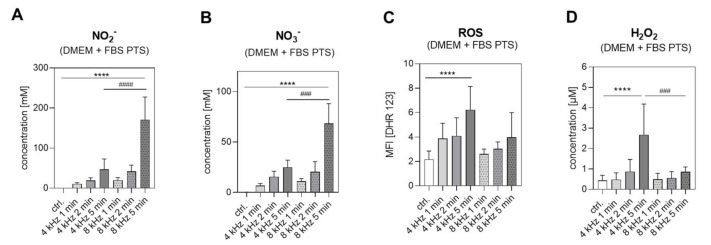
NO_2_^−^, NO_3_^−^, ROS and H_2_O_2_ in DMEM + FBS PTS. In total, 2.5 mL of DMEM + FBS was CAP-treated with 4 kHz for 1, 2 and 5 min and with 8 kHz for 1, 2 and 5 min or remained untreated (control); (n = 3 in duplicated form). The (**A**) NO_2_^−^ and (**B**) NO_3_^−^ concentration [mM] in PTS was quantified using a colorimetric Nitrite/Nitrate Assay Kit. (**C**) The median fluorescence intensity (MFI) of DHR 123 was used as an indicator for CAP-induced ROS in PTS. (**D**) Using an H_2_O_2_ standard series, the Fluorometric Hydrogen Peroxide Assay Kit was used to determine the H_2_O_2_ concentration [µM] in PTS. Statistical analysis: Bonferroni’s multiple comparisons test. **** *p* < 0.0001 (4 kHz, 8 kHz versus control), ^###^ *p* < 0.001, ^####^ *p* < 0.0001 (4 kHz versus 8 kHz).

Reactive oxygen and nitrogen species (RONS) are described as the decisive components for the anti-tumor effect of Cold Atmospheric Plasma (CAP). In order to examine which RONS are produced in plasma-treated medium (PTS), long-lived reactive oxygen species (ROS) were determined based on the scaling of Dihydrorhodamine 123 (DHR 123) fluorescence, hydrogen peroxide (H_2_O_2_) and reactive nitrogen species (RNS) by measuring nitrite (NO_2_^−^) and nitrate (NO_3_^−^). These species were analyzed in Dulbecco’s modified Eagle’s medium (DMEM) containing 10% fetal bovine serum (FBS) (DMEM + FBS), first without CAP treatment (control); then after CAP treatment at 4 kHz for 1, 2, and 5 min; and finally after CAP- treatment at 8 kHz for 1, 2 and 5 min. The measurements after the nitrogen treatment mode (8 kHz) showed dose-dependent stimulation effects, mostly on NO_2_^−^ (Figure 1A) and NO_3_^−^ (Figure 1B), whereas the dose-dependent induction of ROS (Figure 1C) and H_2_O_2_ (Figure 1D) was primarily observed after the 4 kHz (oxygen) treatment mode. The concentrations of RONS in the PTS differed mostly after 5 min of CAP treatment with 4 kHz, and after 5 min of CAP treatment with 8 kHz; therefore, all of the further experiments were carried out with 5 min of CAP treatment.

### 2.2. Amino Acid Concentrations in DMEM +/− FBS PTS

**Figure 2:**
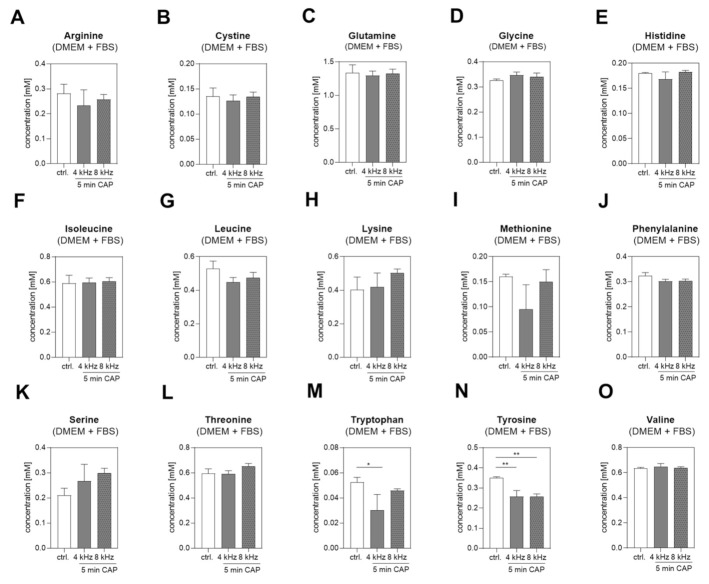
Amino acid concentrations in DMEM + FBS PTS. The concentrations of 15 amino acids (**A**–**O**) were measured by NMR spectroscopy in untreated DMEM + FBS (control), and after 5 min of 4 kHz and 8 kHz CAP treatment (n = 3). Statistical analysis: Bonferroni’s multiple comparisons test. * *p* < 0.05, ** *p* < 0.01 (4 kHz, 8 kHz versus control).

**Figure 3:**
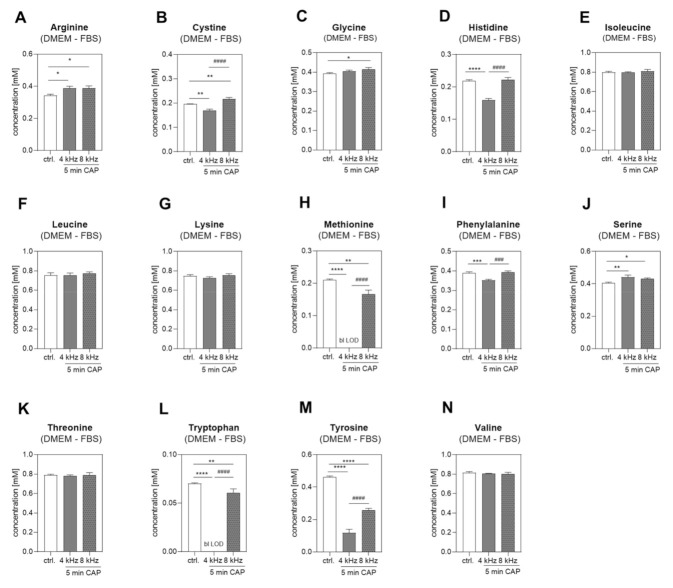
Amino acid concentrations in DMEM–FBS PTS. The concentrations of 14 amino acids (**A**–**N**) were measured by NMR spectroscopy in untreated DMEM–FBS (control), and in DMEM–FBS CAP treated at 4 kHz and 8 kHz for 5 min each (n = 3). Statistical analysis: Bonferroni’s multiple comparisons test. * *p* < 0.05, ** *p* < 0.01, *** *p* < 0.001, **** *p* < 0.0001 (4 kHz, 8 kHz versus control), ^###^ *p* < 0.001, ^####^ *p* < 0.0001 (4 kHz versus 8 kHz); bl LOD: below limit of detection.

First, we analyzed the changes in the amino acid concentrations in DMEM + FBS PTS. The concentration of 15 different amino acids (Figure 2A–O) were determined by NMR spectroscopy before and after 5 min of CAP treatment at 4 kHz and 8 kHz. The Tryptophan (Trp) concentration was significantly decreased after 4 kHz CAP treatment (Figure 2M), and Tyrosine (Tyr) was significantly decreased after both 4 kHz and 8 kHz CAP treatment compared to the control (Figure 2N). Next, 14 different amino acids in DMEM without FBS (DMEM–FBS) PTS were analyzed (Figure 3). Amino acid glutamine was supplemented in the DMEM + FBS medium but not in the DMEM–FBS medium; hence, it was not detectable in the DMEM–FBS medium. Generally, the controls of DMEM + FBS PTS showed lower values, which were obtained because of the dilution with 10% FBS and a varying degree of the protein binding of amino acids. In comparison to DMEM + FBS PTS, additional and more significant changes were observed in the medium without any FBS. The Trp concentration in the DMEM–FBS solution CAP-treated at 4 kHz for 5 min dropped below the detection limit (Figure 3L). The Tyr concentration was also reduced, with a significant and strong reduction in the DMEM–FBS solution CAP-treated at 4 kHz compared to the solution CAP-treated at 8 kHz (Figure 3M). In addition, remarkable changes were observed in the concentration of other amino acids, e.g., Methionine (Met) or Phenylalanine (Phe) (Figure 3H,I). Because of the significant decreases in the concentrations of Tyr and Trp in both DMEM + FBS and DMEM–FBS PTS, we focused on these two amino acids from then on.

### 2.3. Amino Acid Modifications in Trp and Tyr PTS

**Figure 4:**
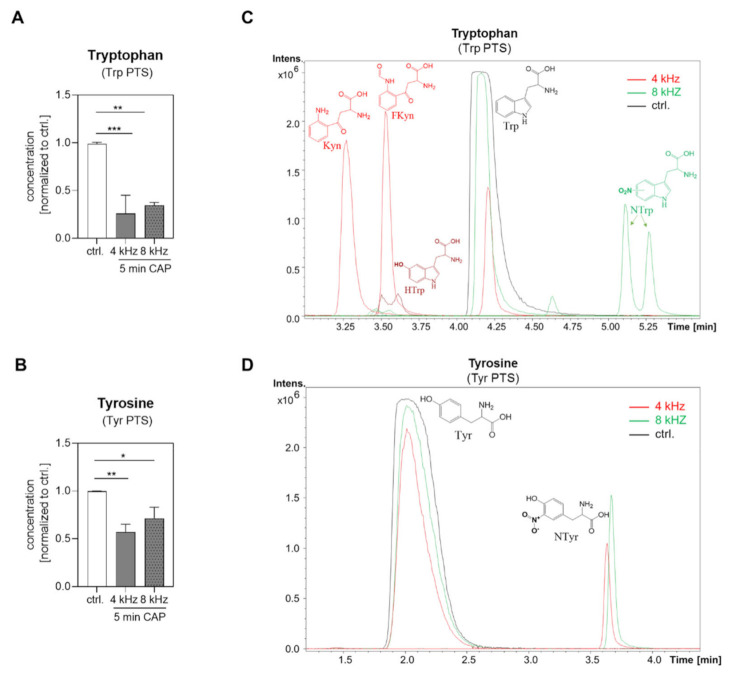
Amino acid modifications in Trp and Tyr PTS. Quantification of the amino acids Trp (**A**) and Tyr (**B**) in Tyr and Trp solution [1 mM in water bidest.] by NMR spectroscopy before CAP treatment (control) and after 5 min of 4 kHz and 8 kHz CAP treatment (n = 3 per solution). The (**C**) HPLC-TOFMS measurements identified HTrp, FKyn, Kyn and NTrp in the Trp PTS, and (**D**) NTyr in the Tyr PTS. All of the compounds were tentatively identified. Statistical analysis: Bonferroni’s multiple comparisons test. * *p* < 0.05, ** *p* < 0.01, *** *p* < 0.001 (4 kHz, 8 kHz versus control).

The pure amino acids Trp and Tyr (1 mM in water bidest.) were investigated with NMR spectroscopy after 5 min of 4 kHz and 8 kHz CAP treatment in comparison to the untreated control (control). As expected, both amino acid concentrations were significantly reduced in Trp and Tyr PTS (Figure 4A,B). HPLC-TOFMS analysis was performed in order to identify the corresponding reaction products. Most of the detected substances, including Hydroxytryptophan (HTrp), Formylkynurenine (FKyn) and Kynurenine (Kyn), were identified in the Trp solution CAP treated at 4 kHz for 5 min, whereas Nitrotryptophan (NTrp) was found in the Trp solution CAP treated at 8 kHz for 5 min (Figure 4C). Furthermore, Tyr was partly converted to Nitrotyrosine (NTyr) with the 4 kHz and 8 kHz CAP-treatment mode (Figure 4D).

### 2.4. Amino Acid Modifications in DMEM–FBS PTS

**Figure 5:**
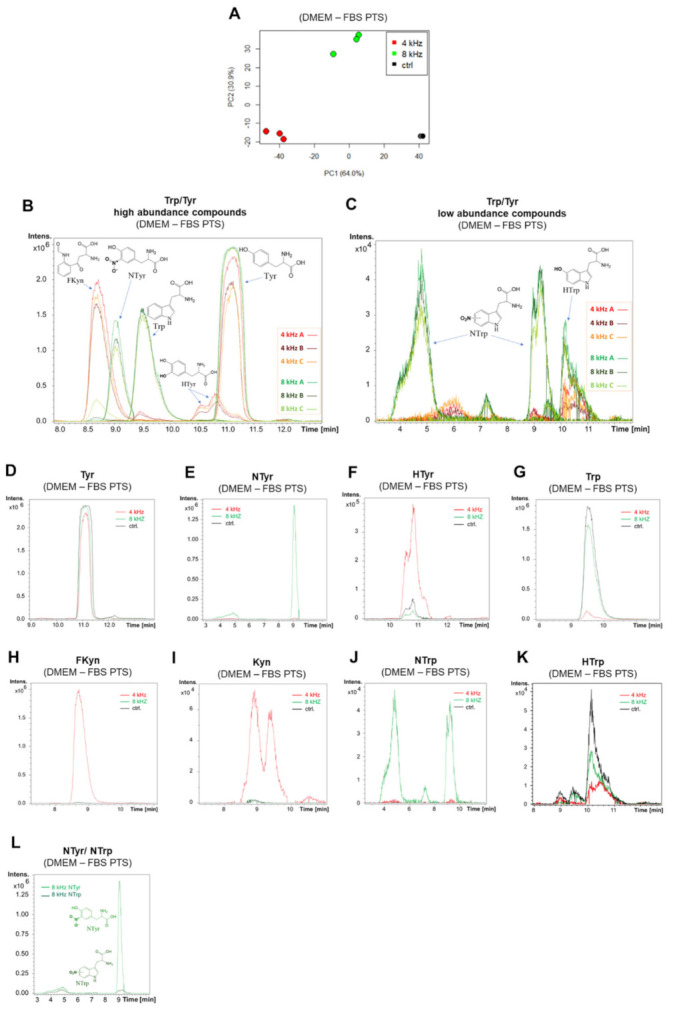
Amino acid degradation in DMEM–FBS PTS. A PCA score plot obtained from the fingerprinting analysis of the HPLC-TOFMS data (**A**). Extracted ion chromatograms (EICs) of the high and low abundance compounds in the triplicates of the 4 kHz and 8 kHz CAP-treated samples (**B**,**C**). EICs of the different metabolites detected in the treated and control samples (**D**–**K**). EIC comparison between the NTrp and NTyr in the same 8 kHz sample (**L**). All of the compounds were tentatively identified.

The analysis of the plasma-treated DMEM–FBS solutions by MS showed further differences. Triplicates of the untreated DMEM–FBS solutions and the DMEM–FBS solutions treated at 4 kHz and 8 kHz for 5 min were analyzed by HPLC-TOFMS using an HILIC column and a positive ionization mode. A fingerprinting analysis using the log_2_ transformed peak areas yielded a PCA plot that showed clear separation among the groups (Figure 5A). Further data evaluation identified the analytes causing the separation. Table S1 shows 29 features that proved to be significant because of an adjusted *p*-value of < 0.05 in the ANOVA test. Figure 5B,C shows the extracted ion chromatograms of the high and low abundant compounds in the DMEM–FBS solutions CAP treated at 4 kHz and 8 kHz for 5 min. In agreement with the PCA plot, each group had comparable chromatograms among its triplicates. The extracted ion chromatograms of Tyr and Trp were compared among the different treatment groups (Figure 5D,G). The NTyr in the 8 kHz group was the most significant product with high abundance (Figure 5E). HTyr existed in the untreated samples and was partially broken down in the 8 kHz mode, but was formed in the 4 kHz mode (Figure 5F). The highest Trp degradation was observed in the 4 kHz groups, with the majority of the Trp being converted to FKyn (Figure 5H), and to a lesser degree to Kyn (Figure 5I), assuming similar ionization effectiveness for these products and no ion suppression. NTrp was formed in the 8 kHz CAP-treated samples (Figure 5J), but with relatively low abundance. Two peaks were observed for NTrp, suggesting two different positions of the nitro functional group. HTrp was broken down in the 4 kHz and 8 kHz CAP-treated samples (Figure 5K). A comparison between the NTrp and NTyr in the same 8 kHz sample showed a significantly higher abundance of NTyr (Figure 5L).

### 2.5. RONS in Tyr and Trp PTS

**Figure 6:**
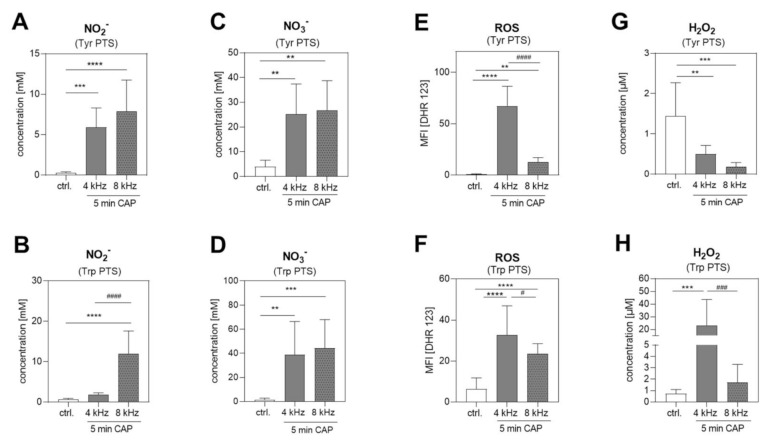
RONS in Tyr and Trp PTS. The RONS in Tyr and Trp PTS were determined after 5 min of 4 kHz and 8 kHz CAP treatment, and were compared to the untreated control (control) (n = 3 in duplicated form). The NO_2_^−^ and NO_3_^−^ concentrations [mM] were measured in Tyr PTS (**A**,**C**) and in Trp PTS (**B**,**D**). The median fluorescence intensities (MFI) of the DHR 123 and H_2_O_2_ concentrations [µM] were determined in Tyr PTS (**E**,**G**) and Trp PTS (**F**,**H**), in comparison to the untreated control. Statistical analysis: Bonferroni’s multiple comparisons test. ** *p* < 0.01, *** *p* < 0.001, **** *p* < 0.0001 (4 kHz, 8 kHz versus control), ^#^ *p* < 0.05, ^###^ *p* < 0.001, ^####^ *p* < 0.0001 (4 kHz versus 8 kHz).

The RONS composition in the Tyr and Trp PTS was analyzed in order to identify possible correlations between plasma-based amino acid modifications and RONS production. The strong induction of NO_2_^−^ (Figure 6A,B) and NO_3_^−^ (Figure 6C,D) was observed in Tyr and Trp PTS produced with the 4 kHz and the 8 kHz CAP treatment mode. A nitrogen (8 kHz) mode dependency of NO_2_^−^ and NO_3_^−^ induction, as observed in DMEM + FBS PTS (Figure 1A,B), was solely observed for NO_2_^−^ in Trp PTS (Figure 6B). ROS production was induced in Tyr and Trp PTS with a significantly higher concentration in the 4 kHz CAP-treated solutions (Figure 6E,F). Remarkably, the H_2_O_2_ concentration was quenched in the Tyr PTS produced with the 4 kHz and the 8 kHz CAP mode compared to the control (Figure 6G), but was elevated (H_2_O_2_ concentration >20 µM) in the 4 kHz CAP-treated Trp PTS (Figure 6H).

### 2.6. pH-Value and Color Analysis of PTS

Because previous studies described CAP-induced acidification as being essential for the cellular effects of PTS [35,36], we determined the pH-value of all of the used PTS. Because of the high buffer capacity of the DMEM medium, no significant pH changes were observed in the DMEM + FBS solutions CAP treated at 4 kHz and 8 kHz for 1 min, 2 min and 5 min compared to the untreated DMEM + FBS medium (Table 1; solution numbers 1–8). Similar results were obtained for DMEM–FBS PTS CAP treated at 4 kHz and 8 kHz for 5 min (Table 1; solution numbers 9–11). In aqueous Tyr and Trp PTS, however, the pH-value dropped significantly in comparison to the untreated control (Table 1; solution numbers 12–17).

Interestingly, a strong color change was noticed in some of the PTS. Plasma dose-dependent coloration was observed in all of the DMEM-containing PTS compared to the corresponding untreated control (Figure S1; solution 1–11), and in the Trp solution CAP treated at 4 kHz for 5 min (Figure S1; solution 16). The ultra-violet/visible (UV/VIS) spectra (Figure S1) of the CAP-treated solutions showed clear differences, potentially indicating different species or other photochemical or thermal reactions as the cause of the color change.

### 2.7. Molecular and Cellular Anti-Tumor Effects of PTS on Melanoma Cells

The anti-tumor effects of PTS on melanoma cells (Mel Im) were investigated by analyzing the expression of different pro-apoptotic and senescence-associated genes (Caspase-3, -7, -9, p16, p21), with the mRNA expression of H2AX as an indicator for DNA damage, apoptosis and senescence. Figure S2 outlines the plasma treatment scheme of the used solutions, all of the treatment groups of melanoma cells with PTS, and the subsequent molecular and cellular analysis. All of the DMEM-containing solutions were supplemented with 10% FBS, 1% P/S and 1% L-glutamine, and the Tyr and Trp solutions were diluted in DMEM with 10% FBS, 1% P/S and 1% L-glutamine (1:1, *v*:*v*) immediately before the cell treatment in order to compensate for CAP-induced pH-value changes, and to optimally nourish the cells during the subsequent incubation.

#### 2.7.1. Molecular Anti-Tumor Analysis of PTS

**Figure 7:**
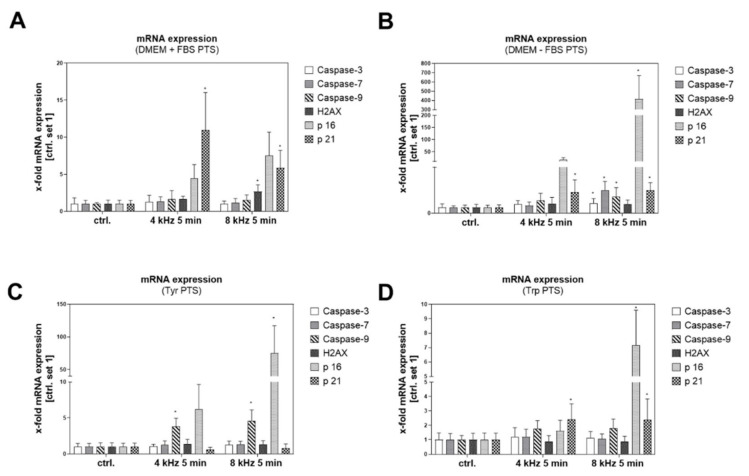
Expression of apoptosis- and senescence-associated genes in Mel Im cells after PTS treatment. The x-fold mRNA expression of Caspase-3, -7, -9, H2AX, p16 and p21 in melanoma cells after 5-min treatment with 4 kHz and 8 kHz CAP solutions was analyzed in comparison to the untreated control (n = 3 in duplicated form). (**A**) DMEM + FBS PTS, (**B**) DMEM–FBS PTS, (**C**) Tyr PTS, (**D**) Trp PTS. Statistical analysis: Bonferroni’s multiple comparisons test. All of the significant results compared to the untreated control are indicated with * *p* ≤ 0.05. The significant results between the 4 kHz and 8 kHz CAP-treated samples are not indicated.

Apoptosis-related and senescence-associated genes were induced in almost all of the PTS-treated samples compared to the corresponding untreated controls (Figure 7A–D), with the strongest x-fold mRNA induction in DMEM–FBS PTS. In contrast to the elevated expression of p16 and p21 in almost all of the PTS groups, the expression of Caspases and H2AX was less affected. Interestingly, the induction of p16, the most important experimental marker that is strongly expressed in the molecular mechanism of cellular senescence, was elevated in all of the 8 kHz CAP-treated solutions in comparison to the 4 kHz CAP-treated samples. For a better comparison, all of the mean values and their standard deviations (SD) are summarized in a separate table (Table S2).

#### 2.7.2. Cellular Anti-Tumor Analysis of PTS

**Figure 8:**
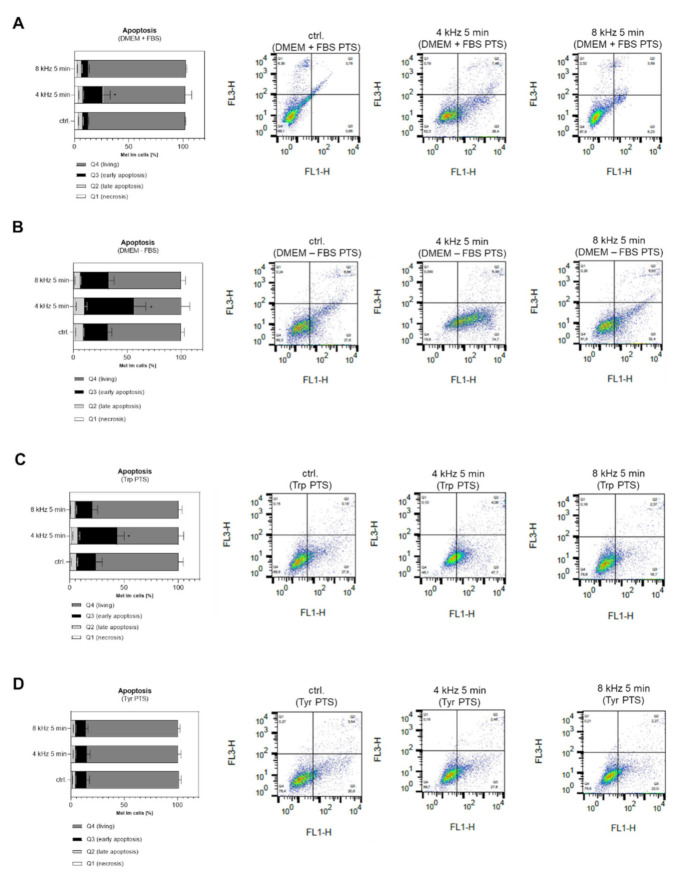
Apoptosis analysis in Mel Im melanoma cells after treatment with PTS. Necrosis (Q1), late apoptosis (Q2), early apoptosis (Q3) and the amount of living cells (Q4) were analyzed in Mel Im cells using FACS PI/ Annexin V analysis 48 h after incubation in DMEM + FBS PTS (**A**), DMEM–FBS PTS (**B**), Trp PTS (**C**) and Tyr PTS (**D**), each CAP treated at 4 kHz and 8 kHz for 5 min, in comparison to the corresponding untreated control solution (control) (n = 3 in duplicated form). The y-axis (FL3-H) shows the PI-labelled population, and the x-axis (FL1-H) shows the FITC-labelled Annexin V positive cells. The necrotic cells are Annexin V negative and PI positive (top-left sector), the apoptotic dead cells are both PI and Annexin V positive (top-right sector), and the apoptotic cells are Annexin V positive and PI negative (lower-right sector). The plots show a typical result from three independent experiments. The graphs present the percentage (mean +/− SD) of the cells in the region among the total cells from three independent experiments in duplicated form. Statistical analysis: Bonferroni’s multiple comparisons test. * *p* < 0.05 (4 kHz, 8 kHz versus control).

Early apoptosis was induced in Mel Im cells after treatment with PTS using DMEM + FBS PTS (Figure 8A), DMEM–FBS PTS (Figure 8B) and Trp PTS (Figure 8C). Interestingly, induction was only achieved with the 4 kHz CAP treatment mode. Tyr PTS did not induce apoptosis in melanoma cells at all (Figure 8D). In addition, apoptosis was investigated after PTS treatment in normal human fibroblasts (NHF) and normal human epidermal melanocytes (NHEM). Here, we also could not observe any apoptosis effect of PTS generated with the 4 kHz and the 8 kHz mode for 5 min (Figure S3A,B). These results support the selectivity of CAP toward tumor cells [37,38].

**Figure 9:**
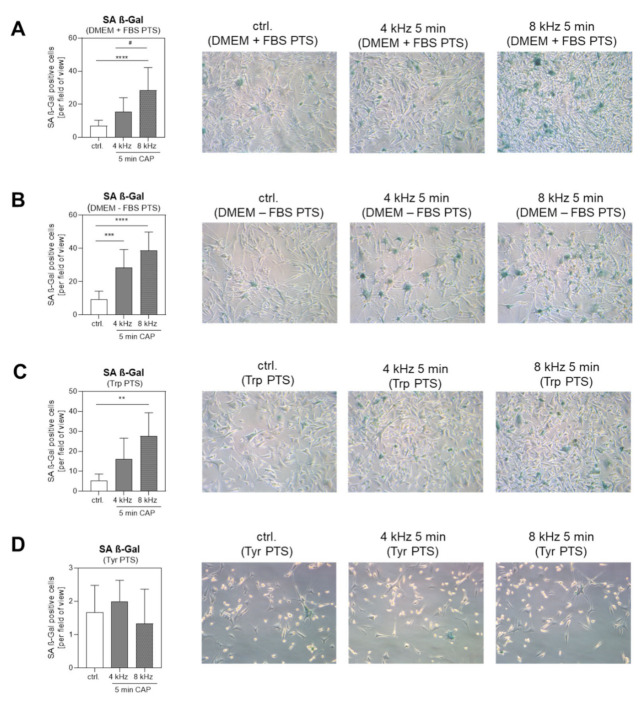
Senescence analysis in Mel Im melanoma cells after treatment with PTS. Light microscopy of Senescence-Associated ß-Galactosidase (SA ß-Gal) staining in melanoma cells 48 h after treatment with DMEM + FBS PTS (**A**), DMEM–FBS PTS (**B**), Trp PTS (**C**) and Tyr PTS (**D**), each CAP treated at 4 kHz and 8 kHz for 5 min, in comparison to the corresponding untreated control solution. The graphs present the percentage (mean +/− SD) of SA ß-Gal positive cells counted in five fields of view (10-fold magnification) from three independent experiments. Statistical analysis: Bonferroni’s multiple comparisons test. ** *p* < 0.01, *** *p* < 0.001, **** *p* < 0.0001 (4 kHz, 8 kHz versus control), ^#^ *p* < 0.05 (4 kHz versus 8 kHz).

The number of Senescence-Associated ß-Galactosidase (SA ß-Gal)-positive cells after treatment with DMEM + FBS PTS (Figure 9A), DMEM–FBS PTS (Figure 9B) and Trp PTS (Figure 9C) was increased in comparison to the corresponding control, with a significantly stronger induction in the 8 kHz CAP-treated solutions than in the 4 kHz CAP-treated samples. The number of SA ß-Gal positive cells was not increased in the Tyr PTS-treated groups (Figure 9D). In addition, senescence was investigated after PTS treatment in normal human fibroblasts (NHF) and normal human epidermal melanocytes (NHEM). Here, we could not observe any significant changes in the SA ß-Gal positive cells after the treatment with PTS generated with the 4 kHz and the 8 kHz mode for 5 min (Figure S3C,D). These results further support the selectivity hypothesis of CAP toward tumor cells [38].

## 3. Discussion

Plasma-treated solutions (PTS) for the treatment of tumor cells are an important new approach with confirmed anti-tumor capacity, which have the potential to be therapeutically effective [22,23,24,25,39]. Elevated levels of ROS, particularly elevated levels of H_2_O_2_, are well known to be lethal to tumor cells [40]. The generation of NO_2_^−^ and NO_3_^−^, and their anti-tumor effects, are also a well-analyzed part of CAP treatment [41]. However, because of the large variety of plasma devices, gas supplies and plasma-treated solutions, it is necessary to detect and quantify the reactive species in the context of the experimental setup. With the prototype version of the plasma care^®^ device, it was possible to produce plasma mode-dependent RONS. The 4 kHz oxygen mode resulted in a significant increase in long-lived ROS and H_2_O_2_ production, whereas the 8 kHz nitrogen mode rather promoted the generation of NO_2_^−^ and NO_3_^−^ in PTS (Table 2).

Our results of the µM to mM ranges of RONS production were supported by studies on PTS using other plasma devices [41,42,43], and displayed an important baseline for the comparison of the CAP effects. Interestingly, these mode-dependent differences in RONS production were neither consistently seen in the Tyr PTS nor in the Trp PTS. The strong H_2_O_2_ quenching was remarkable in the 4 kHz CAP-treated Tyr PTS in comparison to the control, and the strong H_2_O_2_ production (>20 µM) in the 4 kHz CAP-treated Trp PTS. The high concentration of H_2_O_2_ produced with the 4 kHz CAP mode in the Trp solutions may support the observed apoptosis effect in melanoma cells, as already mentioned in other studies [40,42,43,44,45]. The quenched concentration of H_2_O_2_ in Tyr PTS (Figure 6G), however, may prevent the anti-tumor effects, as observed in this study (Figure 8D). In addition, the still-unexplored mechanism leading to the coloration of PTS, which—according to our analyses so far—is related but not limited to the composition of RONS and subsequent Trp modifications, may affect the molecular and cellular behavior of tumor cells. Next to the composition of RONS, the thermal and photochemical reactivity of the Trp metabolite Kyn into Kyn Yellow [46,47,48,49] may also be involved in the coloration of the PTS. Kyn Yellow is one of the 29 significant features identified in the HPLC-TOFMS analysis (Table S1). So far, no studies have investigated the formation of Kyn Yellow in connection with CAP treatment; thus, further investigations are required in this context.

The modifications of amino acids in PTS described in the literature [32,50] provide information for the elucidation of the mechanism of protein inactivation or activation, but do not fully explore its resulting effects on tumor cells. We observed the plasma mode-dependent hydroxylation and nitration of the aromatic rings in Tyr and Trp. Similar chemical modifications have already been observed in solutions treated with CAP [32]. In addition, the reactive oxygen mode triggered the degradation of Trp into the metabolites FKyn and Kyn, whereas the reactive nitrogen mode rather promoted the nitration of Tyr and Trp, suggesting that, in addition to RONS, certain amino acid modifications in PTS contribute to the anti-tumor effect.

Tanaka et al. reported that PTS, which contains amino acids, down-regulates multiple cell survival and proliferation-signaling pathways, including PIK/AKT and Ras/MAPK signaling pathways [51]. In addition, our results clearly showed mode-dependent anti-tumor effects on melanoma cells. Apoptosis was induced in Mel Im cells treated with PTS using a CAP frequency of 4 kHz, whereas PTS produced with the 8 kHz nitrogen mode induced molecular and cellular mechanisms of senescence. Previous studies with another SMD-based plasma source (MiniFlatPlaSter) have already shown plasma dose-dependent anti-tumor effects ranging from senescence to apoptosis [52]. In this study, we were able to further clarify these observations. Modifications of Trp generated with the 4 kHz CAP-mode (e.g., FKyn, Kyn formation; Table 2) promoted apoptosis. However, in this context, it is difficult to dissect the individual effects of FKyn and Kyn on apoptosis and how they act together with the increased concentrations of H_2_O_2_ that were also observed for Trp-only-based PTS in the active oxygen mode. Trp modifications produced with the 8 kHz CAP-mode (e.g., NTrp, NTyr formation) rather affected the mechanism of senescence. A comparison between the NTrp and NTyr formation in the same 8 kHz-treated DMEM–FBS PTS showed the significantly higher abundance of NTyr, suggesting that the nitration of Trp played a rather subordinate role in inducing senescence. The metabolites which ultimately promote the observed anti-tumor mechanisms must be analyzed in further studies. However, HPLC-TOFMS analysis identified 29 significant features (Table S1) that may participate in the promotion of CAP-induced anti-tumor mechanisms.

Other authors have already described a significantly weaker anti-tumor effect of PTS than that of the direct CAP treatment of cells [27]. We observed similar results in comparison to the direct CAP treatment of tumor cells with the plasma care^®^ device that are not yet published. One reason for the weaker anti-tumor effect of PTS in comparison to the direct CAP treatment of cells may be the RONS scavenging when plasma-treated cell culture media are used for cancer cell treatment [53]. Therefore, it is important for the future to determine the effects of plasma and its chemistry on various types of solutions other than media or water, in order to improve the anti-tumor efficacy. Another important reason for the stronger anti-tumor effect of direct CAP treatment could be physical. Next to RONS production, CAP also generates thermal- and ultraviolet (UV) radiation, which also have a strong impact on tumor cell behavior [54,55,56], and which are not available in the case of indirect treatment with PTS.

Even though we observed weaker anti-tumor effects when using PTS than after direct CAP treatment, it is worthwhile to note that a significant induction of early apoptosis and the induction of Caspase-3, -7, -9, H2AX, p16 and p21 were observed after PTS treatment (Figure 7), whereas late apoptosis with cell death was not significantly induced in the present study (Figure 8). Interestingly, the induction of early apoptosis and pro-apoptotic molecules is strongly dependent on the PTS used. Thus, the treatment of Mel Im cells with DMEM-FBS PTS resulted in a stronger induction of caspases-3, -7 and -9 compared to the treatment with DMEM + FBS PTS. In this regard, Tyr PTS led to a significant induction of caspase-9. However, there are pro-apoptotic genes such as H2AX and p21 that are more strongly induced in melanoma cells after DMEM + FBS PTS treatment. Here, the tumor suppressor gene p21 is significantly increased and more strongly expressed using the 4 kHz mode for PTS production. The cause of the strong and striking inductions of p21, primarily observed after DMEM + FBS PTS treatment, needs to be investigated in further studies. However, it is known that p21 is induced by CAP in various tumor studies, and is significantly involved in the anti-tumor effect of CAP [13,14,18,52,57,58]. One of the most striking—and in our opinion, most interesting—results of the expression analysis in this study was the induction of p16 after the PTS treatment. The tumor suppressor p16 (INK4A/MTS-1/CDKN2A) has gained widespread importance in cancer research, because the loss of p16 is described as an early event in cancer progression [59]; however, its induction is strongly associated with the induction of senescence [60]. In the present study, p16 was strikingly induced in Mel Im cells treated with 8 kHz-generated PTS, and correlated more or less with the senescence induction. It is due to this constant regulation among different 8 kHz-generated PTS (DMEM +/− FBS, Tyr PTS and Trp PTS), that let us speculate, that nitrogen species and other 8 kHz-specific chemical changes such as NTyr or NTrp production might be involved in p16 induction; however, further studies are needed to clarify this.

When plasma-treated cell culture media such as DMEM are used for cancer cell treatment, the presence of amino acids, FBS and pyruvate have an impact on the final composition of the PTS and the rate of RONS produced [41,61,62]. These results are in line with our observations. Comparing the molecular and cellular effects on tumor cells after treatment with DMEM +/− FBS-generated PTS, the anti-tumor effects were stronger when the cells were incubated with DMEM–FBS-generated PTS. We would like to point out that this effect is not due to FBS deficiency during the cell treatment, because FBS was supplemented before the cells were treated with PTS. Thus, the observed cellular or molecular effects are due to the different chemical reactions formed during the generation of DMEM PTS with or without the addition of FBS. More precisely, after the treatment of the cells with DMEM–FBS PTS, we detected significantly more and stronger amino acid modifications, as well as the stronger expression of apoptosis- and senescence-associated genes and the induction of apoptosis and senescence than after cell treatment with DMEM + FBS PTS.

In conclusion, the use of plasma-treated solutions (PTS) to kill tumor cells is an effective adjunct to direct the CAP treatment of malignant cells that has great potential, especially for the treatment of internal tumors or metastases. Next to the plasma mode-dependent production of RONS in PTS, mode-dependent amino acid degradation and modification, which alter the molecular and cellular behavior of tumor cells, are of great importance. Trp metabolism appears to play a crucial role in the anti-tumor effect of PTS. Depending on the plasma mode and the formation of specific Trp metabolites, mechanisms such as senescence or apoptosis can be triggered. How these metabolites are taken up by the cells and influence the cellular metabolism is a complex field, and a point of interest for future research; the more the entire potential of PTS and its anti-tumor effect is elucidated, the more effective may be the development of tumor therapeutic approaches. The next step will be to evaluate 4 kHz and 8 kHz generated PTS in a melanoma mouse model in order to analyze its effect and efficacy on different cells in a biological system.

## 4. Materials and Methods

### 4.1. Plasma Source

A prototype of the plasma care® device (Terraplasma GmbH, Garching, Germany) was used for CAP treatment. The design, technology and ozone spectrum of the device was recently published [63]. This prototype enables changes in frequency between an oxygen (4 kHz) and a nitrogen (8 kHz) mode. This device uses a technology termed “thin-film technology”, a further development of surface micro-discharge technology (SMD) [52]. The use of high voltage of 3.5 kV provokes millimeter-sized micro-discharges into the plasma-source unit. This unit consists of a high-voltage electrode, and a dielectric and a grounded structured electrode, which subsequently produce plasma components alterable by frequency and voltage. In order to guarantee a standardized distance between the device and the 35 mm petri dish (Corning; Merck, Darmstadt, Germany), the device was placed onto a spacer [63], simultaneously ensuring an isolated treatment area. The production of PTS with the plasma care® device took place under standardized conditions in a climate-controlled room, where a maximum of 5% climate fluctuation is measured in order to make sure that exogenous factors such as humidity and temperature do not affect the production of RONS.

### 4.2. Preparation of the Plasma-Treated Solutions (PTS)

In order to prepare the plasma-treated solutions (PTS), 2.5 mL liquid was placed into a 35 mm petri dish (Corning; Merck, Darmstadt, Germany), together with a magnetic stir bar stirring at 300 rounds per min (rpm). If not otherwise noted, all of the liquids were treated at a frequency of 4 kHz or 8 kHz for 5 min; the corresponding control liquids remained untreated. The following liquids were used: Dulbecco’s modified Eagle’s medium (DMEM) (ThermoFisher Scientific, Schwerte, Germany) with 10% fetal bovine serum (FBS) (Anprotec, Bruckberg, Germany), 1% penicillin/streptomycin (P/S) and 1% L-glutamine (Sigma Aldrich GmbH, Steinheim, Germany) (abbreviation: DMEM + FBS), DMEM without any additives (abbreviation: DMEM–FBS), aqueous solutions of Tyrosine (Tyr) and Tryptophan (Trp) (1 mM in water; Sigma Aldrich GmbH, Steinheim, Germany), and melanocyte growth medium (MGM; CC-3249; Lonza Group Ltd, Basel, Switzerland) exclusively for the treatment of NHEM. The water used to prepare the solutions and the samples for the NMR and HPLC-TOFMS measurements was purified using a PURELAB Plus system (ELGA, LabWater, Celle, Germany).

### 4.3. Assessment of the ROS, H_2_O_2_ and NO_2_^−^/NO_3_^−^ in PTS

For the ROS detection, a final 10 µM solution of Dihydrorhodamine 123 (DHR 123, Sigma Aldrich GmbH, Steinheim, Germany) was incubated with 100 µL PTS immediately after the CAP treatment in a black 96-well plate (Greiner Bio-One GmbH, Frickenhausen, Germany). The fluorescence was detected at an excitation wavelength of 505 nm and an emission wavelength of 534 nm. For the quantification of the H_2_O_2_, a Fluorimetric Hydrogen Peroxide Assay Kit (Sigma Aldrich GmbH, Steinheim, Germany) was used, and the fluorescence was measured at an excitation wavelength of 540 nm and an emission wavelength of 590 nm. The NO_2_^−^ and NO_3_^−^ concentrations were determined using a colorimetric Nitrite/Nitrate Assay Kit (Sigma Aldrich GmbH, Steinheim, Germany) to detect the nitric oxide metabolites at 540 nm absorbance. The fluorescence was measured with a plate reader (Varioscan Flash, Thermo Fisher, Schwerte, Germany), and the assay kits were used as specified by the manufacturer.

### 4.4. pH-Value Measurement of the PTS

The pH-value was measured in the PTS with a pH meter immediately after the plasma treatment (Mettler-Toledo GmbH, Giessen, Germany). For this purpose, the PTS was transferred from a 35 mm petri dish into a 5 mL tube (Greiner Bio-One GmbH, Frickenhausen, Germany).

### 4.5. Color Analysis of the PTS

The coloration of the PTS was documented by photography, determined with a spectrophotometer (Specord 50 Plus; Analytik Jena AG, Jena, Germany) and analyzed with WinASPECT PLUS software (Analytik Jena AG, Jena, Germany).

### 4.6. Determination of the Amino Acids in the PTS by NMR Spectroscopy

PTS in triplicates were transferred into 5 mL tubes (Eppendorf, Hamburg, Germany) and mixed overhead. Then, 400 µL of the unfiltered sample was placed in an NMR tube (Bruker BioSpin GmbH, Rheinstetten, Germany) with 250 µL NMR-H_2_O buffer (8.02 mL 1M K_2_HPO_4_ (Carl Roth GmbH, Karlsruhe, Germany), 1.98 mL 1M KH_2_PO_4_ (Merck, Darmstadt, Germany), 90 mL H_2_O, 30 mg Borat (Merck, Darmstadt, Germany), and 25 mL D_2_O with 0.75 wt. % TSP (Sigma Aldrich GmbH, Steinheim, Germany). In the samples containing FBS, 10 µL formic acid (81.97 mM; Merck, Darmstadt, Germany) was added as an additional internal standard, because formic acid is not prone to protein binding. The NMR was measured with a Bruker Avance III 600 MHz spectrometer employing a triple-resonance (^1^H, ^13^C ^15^N, ^2^H lock), He-cooled cryogenic probe equipped with z-gradients and an automatic sample changer. For each sample, the probe was automatically locked, tuned, matched and shimmed. All of the spectra were measured at 298 K, and each sample was allowed to equilibrate for 300 s in the magnet before its measurement. The temperature unit was calibrated using a deuterated methanol sample. Spectra of 1D ^1^H of each sample were automatically collected using the Bruker automated acquisition suite ICON-NMR. All of the spectra were acquired without spinning. 1D ^1^H NMR spectra were obtained using a 1D NOESY pulse sequence with presaturation during the relaxation and mixing time and additional spoil gradients for water suppression. For each spectrum, 128 scans were collected into 72k data points using a relaxation delay of 4 s and an acquisition time of 3.07 s. Employing TopSpin 3.1 (Bruker BioSpin GmbH, Rheinstetten, Germany), the spectra were Fourier transformed and phase corrected, applying a line broadening of 0.3 Hz and zero-filling to 128 k points. The spectra were baseline-corrected by applying a polynomial baseline correction.

### 4.7. Metabolite Quantification from the NMR Spectra

The amino acids in the PTS were quantified in relation to the TSP (-FBS) or formic acid (+FBS) reference signal. The accurate determination of the peak integrals, even from partially overlapping signals, required spectral deconvolution. For this purpose, the CHENOMX 8.1 (Chenomx Inc., Edmonton, AB, Canada) software suite was used. First, automatically fitted integrals were obtained, which were checked manually.

### 4.8. Analysis of the Amino Acid Degradation in the PTS by HPLC Time of Flight Mass-Spectrometry (HPLC-TOFMS)

HPLC-TOFMS was performed on a random sample from each triplicate of Tyr and Trp PTS diluted 1:10 with water, and on all of the triplicates of DMEM–FBS diluted 1:5 with water. A Dionex Ultimate 3000 UHPLC system (ThermoFisher Scientific, Idstein, Germany) coupled to a Maxis Impact QTOF-MS (Bruker Daltonics, Bremen, Germany) through an ESI source was employed. For the Tyr and Trp PTS, the chromatographic separation was accomplished with a Kinetex^TM^ column (2.6 µm C18 100 × 2.1 mm id, Phenomenex, Aschaffenburg, Germany) at 35 °C using 0.1% formic acid (Merck, Darmstadt, Germany) in both water (eluent A) and acetonitrile (ACN, eluent B) (Merck, Darmstadt, Germany) at a flow-rate of 0.3 mL/min. The elution was carried out with the following eluent B gradient: 0–40% in 10 min, 40–100% in 2 min, 100% for 5 min, and back to 0% in 0.1 min, followed by equilibration for 8 min. Sample volumes of 5 µL were injected. The analytes in the DMEM–FBS samples were separated on a SeQuant^®^ ZIC^®^-pHILIC column (5 μm, 2.1 mm × 150 mm, Merck, Darmstadt, Germany) at 35 °C using 5 mM ammonium formate in water as eluent A and pure ACN as eluent B, at a flow rate of 0.2 mL/min. The elution was accomplished by the following eluent B gradient: 90–50% in 20 min, 50–40% in 3 min, 40% for 15 min, back to 90% in 5 min, and 17 min equilibration. An injection volume of 5 μL was used.

Electrospray ionization in positive mode was employed using the following settings to operate the source and the mass spectrometer: drying gas, nitrogen with a temperature of 220 °C and a flow rate of 10 L/min; pressure of the nebulizer gas (nitrogen), 2.6 bar; end plate offset, 500 V; capillary voltage, 4500 V; mass range 50–1000 m/z; acquisition rate, 2 spectra/s. Before the measurements, an external calibration of the mass spectrometer was implemented using sodium formate cluster ions (10 mM sodium formate in 50:50 *v*/*v* water/ isopropanol). Additionally, each run included an injection of the sodium formate solution by means of a six-port valve for internal recalibration. A mass spectral resolution of R = 22,000 was obtained. The samples were measured in a random order.

### 4.9. Fingerprinting Analysis

DataAnalysis version 4.1 (Bruker Daltonics, Bremen, Germany) was used for the manual examination and processing of the HPLC-TOFMS chromatograms and mass spectra, the compound extraction, the internal recalibration of the mass spectra, and the calculation of accurate masses. The isotopic pattern fit was evaluated by the calculation of the mSigma value in the SmartFormula tool from Bruker Daltonics. In the case of a perfect match, the mSigma value is 0, whereas a value of 1000 means no match. MSConvert version 3.0.19, which is a tool of ProteoWizard (Palo Alto, CA, USA) [64], was used to convert the files to the .mzxml file format, using the peak picking filter with the parameter “vendor”. MZmine 2 version 2.52 [65] was used for the untargeted analysis, in which the chromatographic peaks were detected, deconvoluted, aligned and filtered. The peak areas were extracted for further evaluation.

For the feature identification, accurate masses were obtained using the sodium formate cluster for internal recalibration, allowing the use of a mass tolerance of only three mDa to limit the number of possible molecular formulae. The isotopic pattern match was evaluated based on the mSigma value in the SmartFormula tool from Bruker Daltonics. Formulae with the lowest mass error and lowest mSigma value were used for tentative identification using the Human Metabolome Database (HMDB).

### 4.10. Cell Culture

The melanoma cell line Mel Im has been described previously [66]. Normal human fibroblasts (NHF; FC-0024) were purchased from Cell Systems GmbH, Troisdorf, Germany, and normal human epidermal melanocytes (NHEM; CC-2504) were purchased from Lonza Group Ltd., Basel, Switzerland. Mel Im and NHF were maintained in DMEM (ThermoFisher Scientific, Schwerte, Germany) and supplemented with 10% FBS (Anprotec, Bruckberg, Germany) and 1% P/S (Sigma Aldrich GmbH, Steinheim, Germany), as well as 1% L-glutamine (Sigma Aldrich GmbH, Steinheim, Germany). The NHEM were cultivated in an MGM-4 Melanocyte Growth Medium-4 BulletKit (MGM; CC-3249; Lonza Group Ltd, Basel, Switzerland). All of the cells were cultured in 75 cm^2^ Falcon^®^ Cell Culture Flasks (Corning Life Sciences, Amsterdam, The Netherlands), incubated under humid conditions in a 5% CO_2_ incubator at 37°C and split 1:3 every 3 days. Following a washing step with Dulbecco’s Phosphate Buffered Saline (DPBS) (ThermoFisher Scientific, Schwerte, Germany), a solution of 0.05% trypsin/0.02% EDTA (Sigma Aldrich GmbH, Steinheim, Germany) in DPBS was applied to detach the cells. After the centrifugation and removal of the trypsin solution, the cells were counted using Luna FL Acridine Orange/ Propidium Iodide Stain (BioCat GmbH, Heidelberg, Germany). Mycoplasma contamination was regularly excluded according to the manufacturer’s instructions of the PCR Mycoplasma Test Kit (PanReac AppliChem, Darmstadt, Germany).

### 4.11. Treatment of the Cells with PTS

In total, 5 × 10^4^ Mel Im or NHF cells were seeded in 2.5 mL DMEM (ThermoFisher Scientific, Schwerte, Germany) with 10% FBS (Anprotec, Bruckberg, Germany), 1% P/S (Sigma Aldrich GmbH, Steinheim, Germany) and 1% L-glutamine (Sigma Aldrich GmbH, Steinheim, Germany) in a 6-well plate (Corning Life Sciences, Amsterdam, The Netherlands) and cultivated for 24 h. The NHEM were seeded in 2.5 mL MGM (Lonza Group Ltd., Basel, Switzerland) and cultivated under the same condition. The medium was removed and replaced with 1 mL PTS. The DMEM–FBS PTS was supplemented with 10% FBS (Anprotec, Bruckberg, Germany), 1% P/S (Sigma Aldrich GmbH, Steinheim, Germany) and 1% L-glutamine (Sigma Aldrich GmbH, Steinheim, Germany) before cell treatment. Tyr PTS and Trp PTS were mixed 1:1 (*v*/*v*) with untreated DMEM supplemented with 10% FBS, 1% P/S and 1% L-glutamine, followed by incubation for a further 24 h (RNA) and 48 h (apoptosis and senescence). Figure S2 summarizes the generation of the PTS, the PTS treatment of the cells, and the subsequent molecular and cellular analysis. Because the cultivation of NHEM in DMEM-containing PTS is not possible, the cells were treated with 1 mL MGM PTS.

### 4.12. Measurement of the Cell Apoptosis

For the analysis of the apoptosis, the cells were treated with PTS as described above. The apoptotic cells were investigated by flow cytometry 48 h after treatment using the FITC Annexin V Apoptosis Detection Kit with PI (BioLegend, Koblenz, Germany) according to the manufacturer’s instructions. The flow cytometry analysis was performed with a FACSCalibur Flow Cytometer (Becton Dickinson, Heidelberg, Germany). The FACS data were analyzed using the FlowJo™ v10 software. The experiments were conducted in duplicates and repeated three times.

### 4.13. Measurement of Cellular Senescence

Senescence-associated ß-Galactosidase (SA ß-Gal) staining was performed 48 h after the PTS treatment according to the manufacturer’s instructions of the Senescence ß-Galactosidase Staining Kit (Cell Signaling Technology, Frankfurt am Main, Germany). From each approach, five images were taken, and the number of positive cells per visual field were determined. The graphs represent a summary of three independent experiments. Representative pictures were collected by light microscopy (Zeiss, Axiovision, Oberkochen, Germany) using a 10-fold magnification.

### 4.14. RNA Isolation and Reverse Transcription

The cellular RNA was isolated 24 h after the treatment with PTS using the NucleoSpin^®^ RNA Kit (Macherey-Nagel, Düren, Germany) according to the manufacturer’s instructions. An average value of 2–5 µg RNA was then transcribed into cDNA by a reverse transcriptase reaction using the Super Script^TM^ II Kit (Invitrogen, Thermo Fisher Scientific, CA, USA).

### 4.15. Quantitative Real-Time PCR Analysis

The gene expression analysis consisted of quantitative real-time PCR with specific sets of primers (Sigma Aldrich GmbH, Steinheim, Germany) and conditions (Table S3) using LightCycler technology (Roche Diagnostics, Mannheim, Germany), as described elsewhere [67]. The PCR reactions were evaluated by melting curve analysis. Beta-actin was amplified to ensure the cDNA’s integrity and to normalize expression. Each experiment was repeated at least three times, in duplicates.

### 4.16. Statistical Analysis

The data were expressed as mean values +/− standard deviation (SD). The data were analyzed with GraphPad Prism 5 software (GraphPad Software Inc., San Diego, CA, USA). The results were analyzed by Ordinary-one-way ANOVA with Bonferroni’s multiple comparisons test. The differences between the untreated and treated samples were considered statistically significant at * *p* < 0.05, ** *p* < 0.01, *** *p* < 0.001 and **** *p* < 0.0001. Significant differences between the 4 kHz and the 8 kHz treatment groups were indicated with ^#^ *p* < 0.05, ^##^ *p* < 0.01, ^###^ *p* < 0.001 and ^####^ *p* < 0.0001.

For the PCA analysis of the MS fingerprinting data, R package version 3.6.1 (http://cran.r-project.org/ accessed 2 Dec 2019) [68] was employed. The missing values were imputed with a tenth of the peak area of the lowest peak area in the sample. The data was log_2_ transformed in order to enforce a normal data distribution, and to reduce heteroscedasticity [69]. The principal component analysis (PCA) was performed with the function prcomp. The significance of the differences in the detected features between the different treatments was determined within R using the limma package [70].

## Figures and Tables

**Table 1:** pH-value in PTS solutions.

Solution Number	Solution Name	pH-Value ^1^ (mean +/− SD)
1	DMEM+FBS (ctrl.)	7.23 +/− 0.32
2	DMEM+FBS (4 kHz 1 min)	7.65 +/− 0.14
3	DMEM+FBS (4 kHz 2 min)	7.76 +/− 0.12
4	DMEM+FBS (4 kHz 5 min)	7.77 +/− 0.09
5	DMEM+FBS (ctrl.)	7.23 +/− 0.32
6	DMEM+FBS (8 kHz 1 min)	7.69 +/− 0.34
7	DMEM+FBS (8 kHz 2 min)	7.79 +/− 0.26
8	DMEM+FBS (8 kHz 5 min)	7.91 +/− 0.29
9	DMEM–FBS (ctrl.)	7.84 +/− 0.31
10	DMEM–FBS (4 kHz 5 min)	7.87 +/− 0.27
11	DMEM–FBS (8 kHz 5 min)	7.93 +/− 0.26
12	Tyrosine (ctrl.)	8.86 +/− 0.09
13	Tyrosine (4 kHz 5 min)	3.66 +/− 0.20 ****
14	Tyrosine (8 kHz 5min)	3.32 +/− 0.10 ****
15	Tryptophan (ctrl.)	6.70 +/− 0.24
16	Tryptophan (4 kHz 5 min)	3.52 +/− 0.19 ****
17	Tryptophan (8 kHz 5min)	3.37 +/− 0.12 ****

^1^ pH-value: mean +/− standard deviation, SD; n = 3. The treatment mode and the treatment duration are indicated within the solution name. Statistical analysis: Bonferroni´s multiple comparisons test. **** *p* < 0.0001 (4 kHz, 8 kHz versus corresponding ctrl.).

**Table 2:** Summary. Mode-dependent CAP effects on PTS and its influence on molecular- and cellular mechanisms in Mel Im melanoma cells.

CAP Treatment Mode	PTS	4 kHz (Oxygen Mode)	8 kHz (Nitrogen Mode)
Duration		5 min	5 min
RONS production	DMEM+FBS DMEM–FBS Trp Tyr	ROS ++, H_2_O_2_ ++ ROS +++, H_2_O_2_ +++ ROS ++, H_2_O_2_ +++ ROS ++, H_2_O_2_ ---	NO_2_ ++, NO_3_ ++ NO_2_ +++, NO_3_ +++ NO_2_ +++, NO_3_ ++ NO_2_ +++, NO_3_ +++
amino acid degradation	DMEM+FBS DMEM–FBS Trp Tyr	Tyr, Trp Cys, His, Met, Phe, Tyr, Trp Trp Tyr	Tyr Tyr, Trp Trp Tyr
amino acid modification	DMEM–FBS Trp Tyr	HTyr, FKyn, Kyn HTrp, FKyn, Kyn NTyr	NTyr, NTrp NTrp NTyr
PTS coloration	DMEM+FBS DMEM–FBS Trp Tyr	++ ++ +	++ ++
p16 gene expression	all PTS	+	+++
apoptosis induction	DMEM+FBS DMEM–FBS Trp Tyr	++ +++ +++	
senescence induction	DMEM+FBS DMEM–FBS Trp Tyr	+ + +	+++ +++ ++

Note: + moderate increase ++ remarkable increase +++/--- strong increase/reduction in comparison to the untreated control.

## Supplementary Materials

The following are available online at https://www.mdpi.com/article/10.3390/ijms22157886/s1.
