# Supplementary figures and images for: Cold Atmospheric Plasma Changes the Amino Acid Composition of Solutions and Influences the Anti-Tumor Effect on Melanoma Cells
Source: Int J Mol Sci. 2021 Jul 23;22(15):7886. doi: 10.3390/ijms22157886 (PMC8346059; doi:10.3390/ijms22157886)

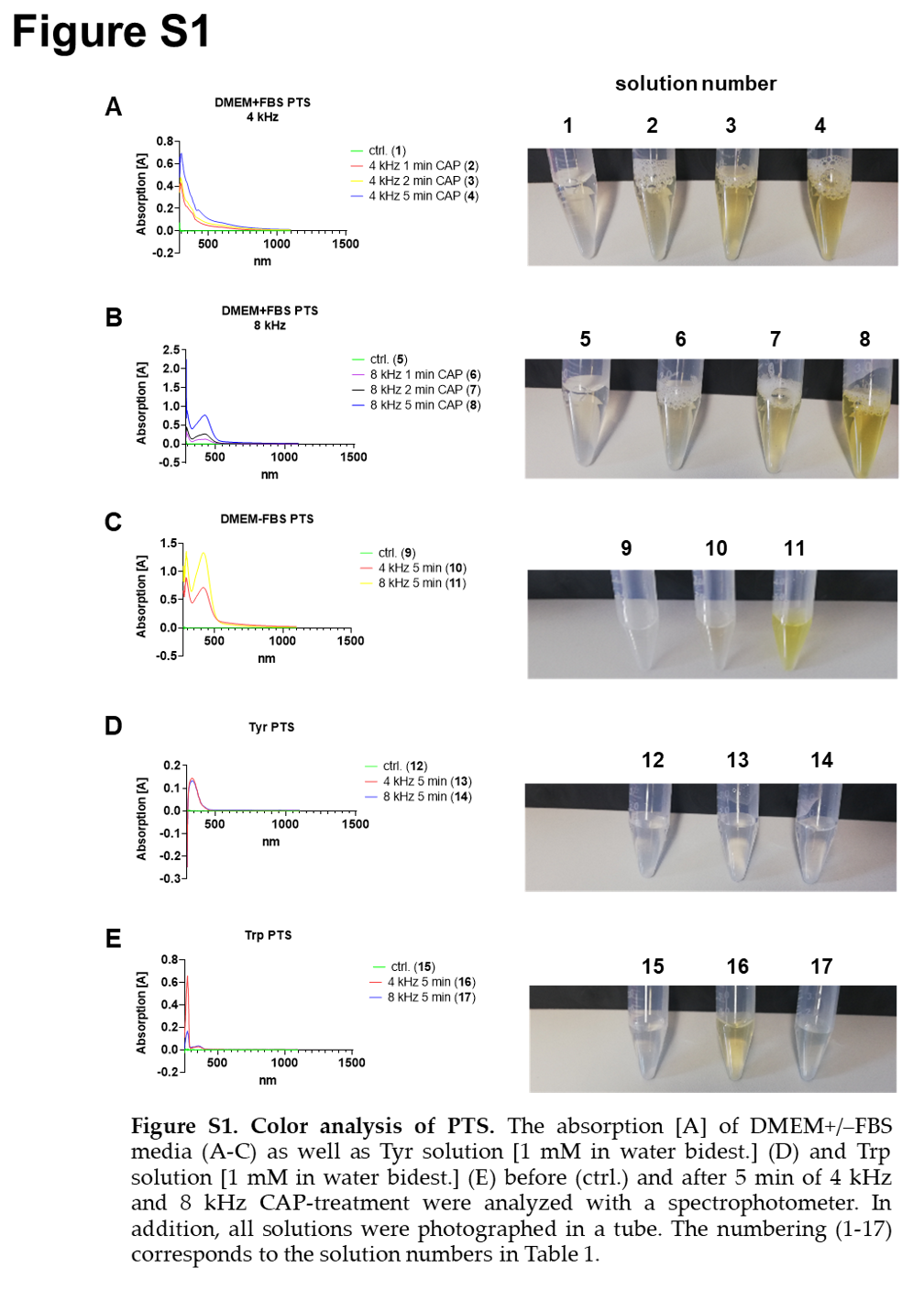

Supplement: Supplementary file 1 [file ijms-22-07886-s001.zip › Figure_S1.tif]

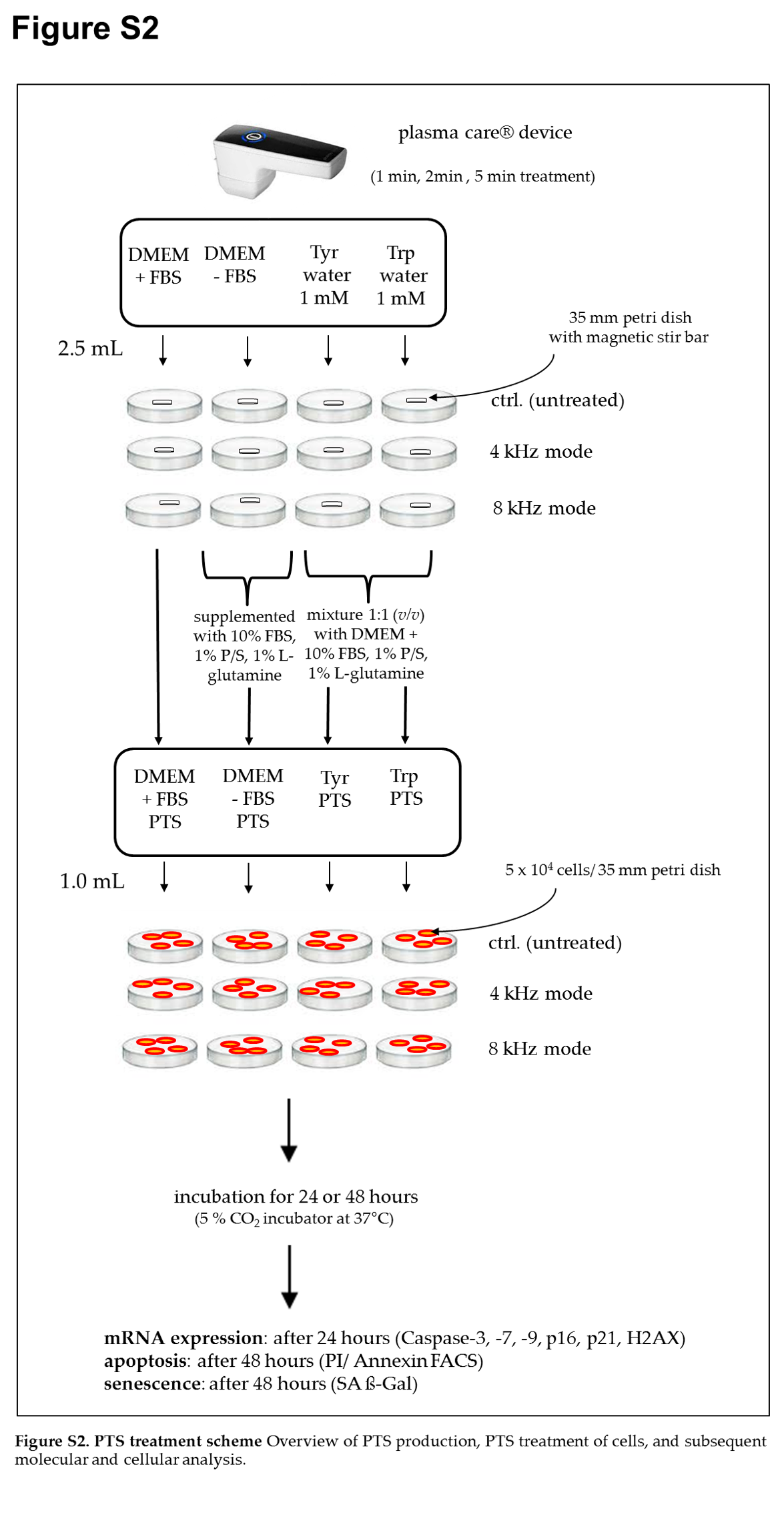

Supplement: Supplementary file 1 [file ijms-22-07886-s001.zip › Figure_S2.tif]

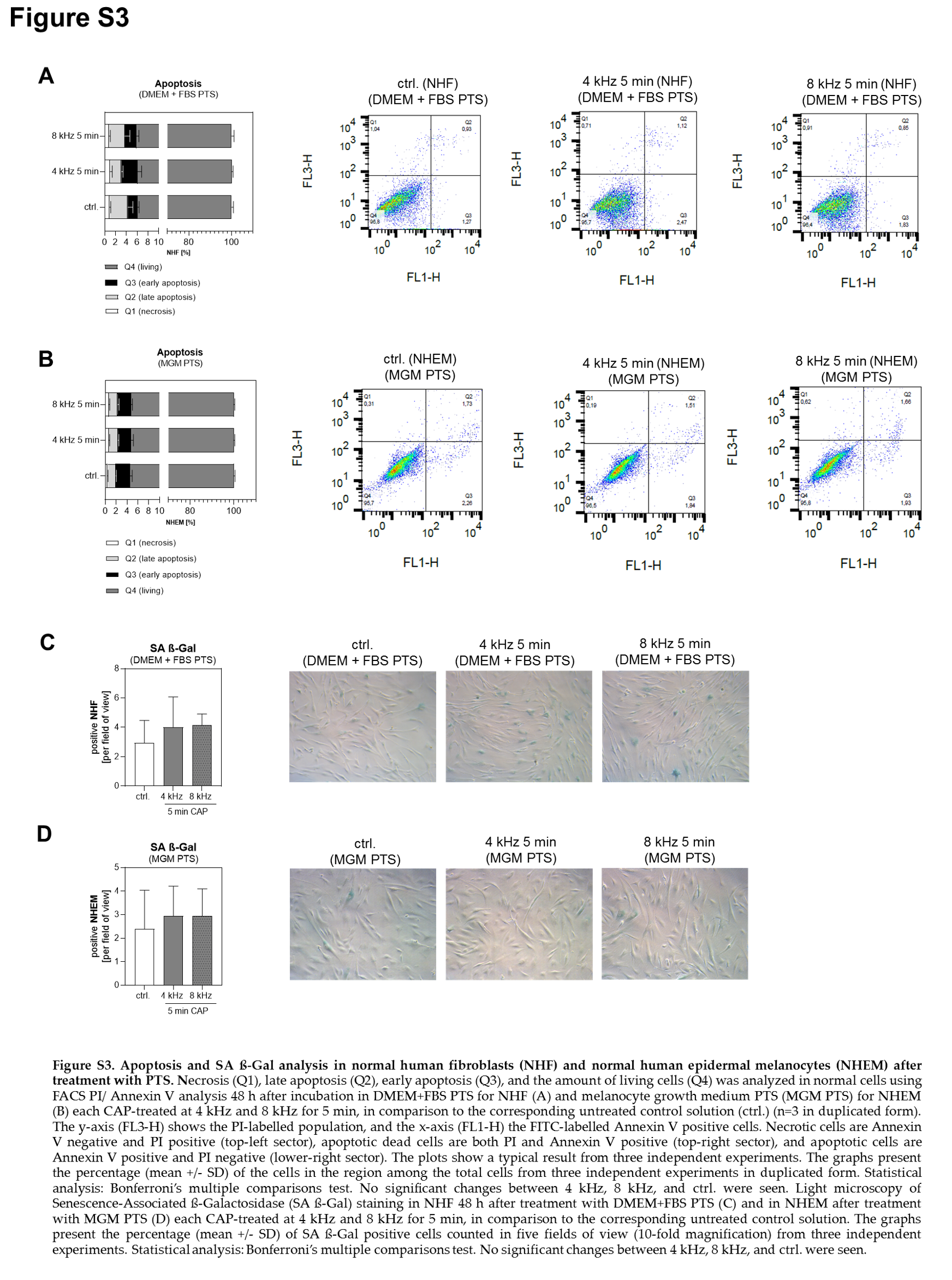

Supplement: Supplementary file 1 [file ijms-22-07886-s001.zip › Figure_S3.tif]

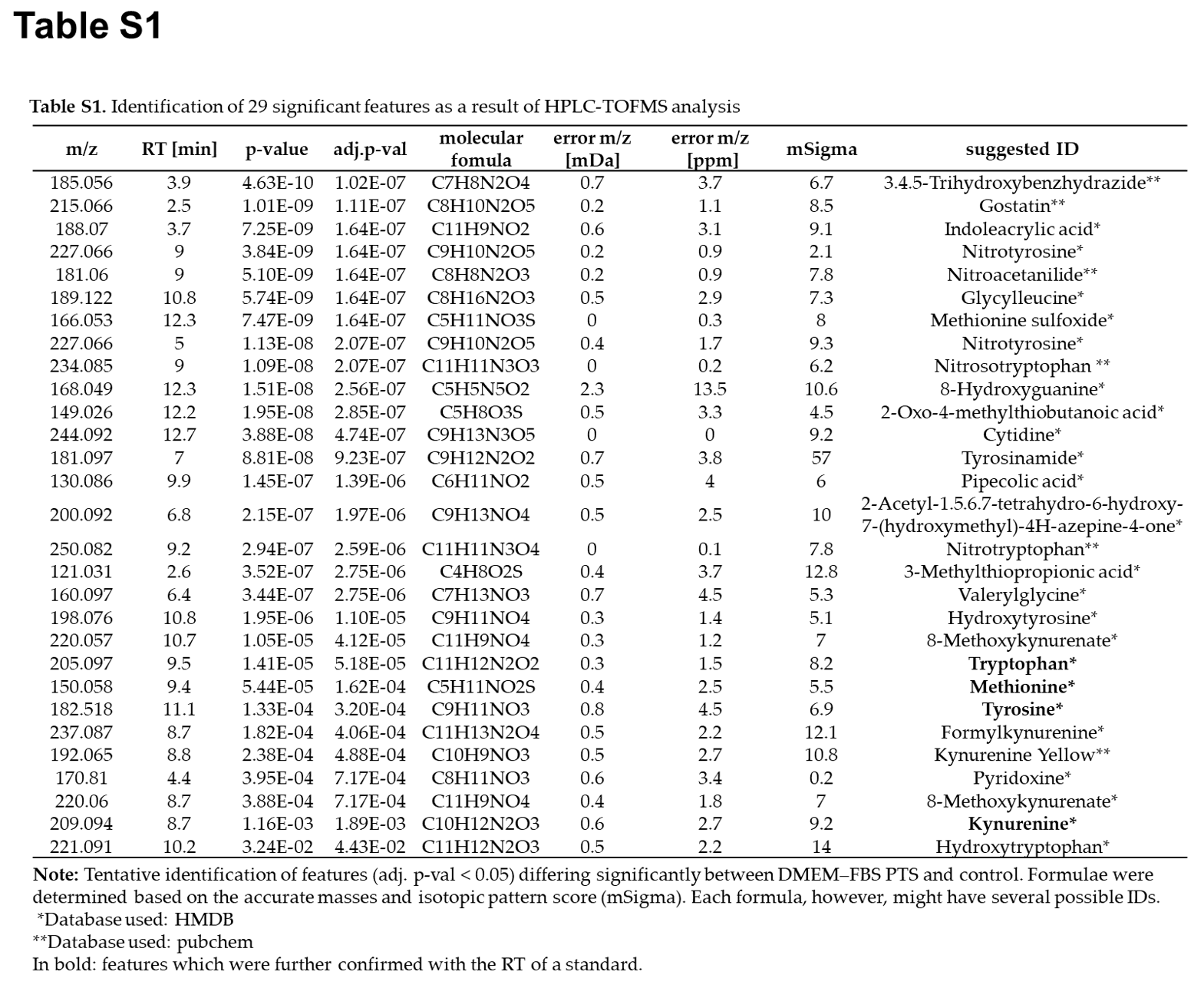

Supplement: Supplementary file 1 [file ijms-22-07886-s001.zip › Table_S1.tif]

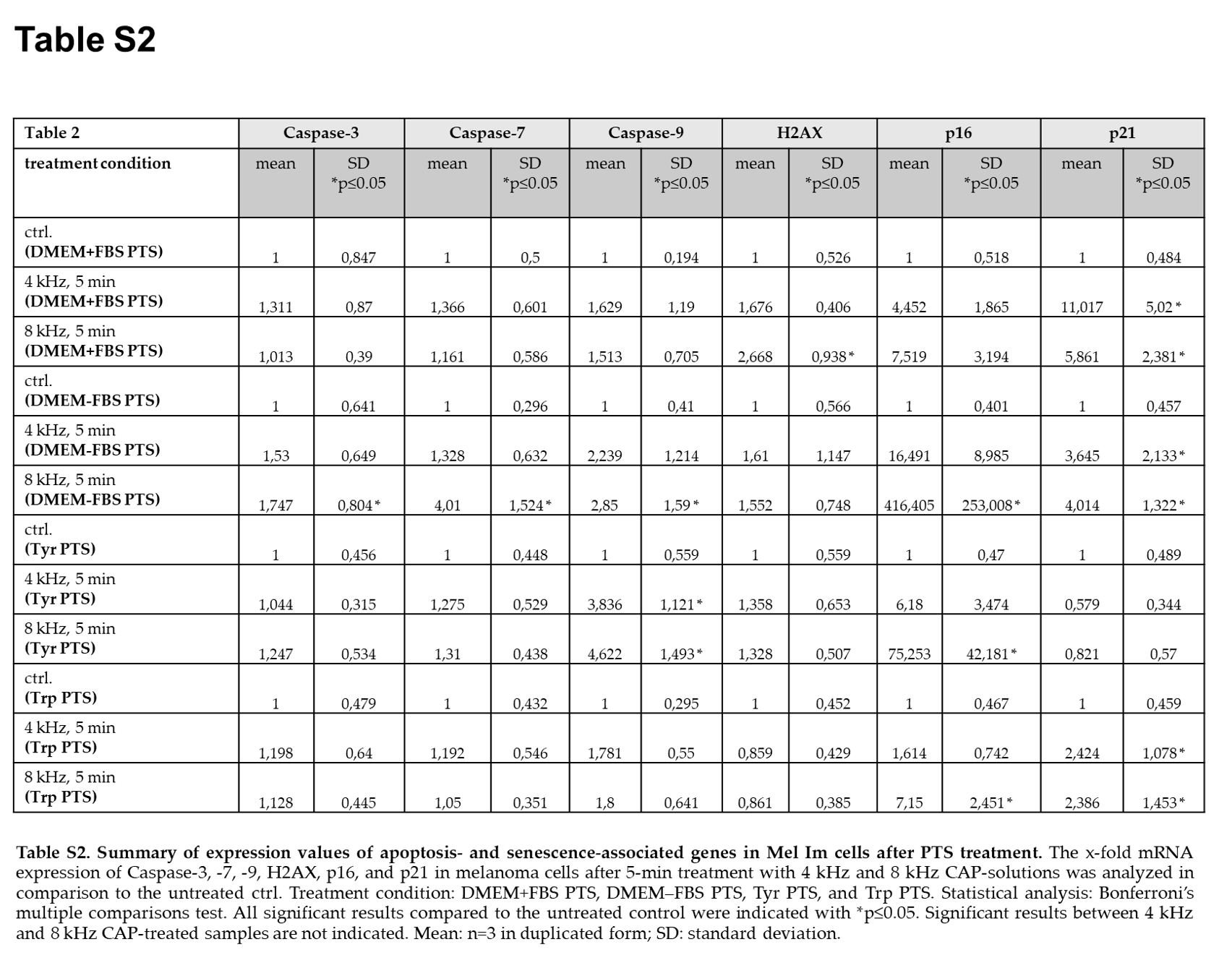

Supplement: Supplementary file 1 [file ijms-22-07886-s001.zip › Table_S2.tif]

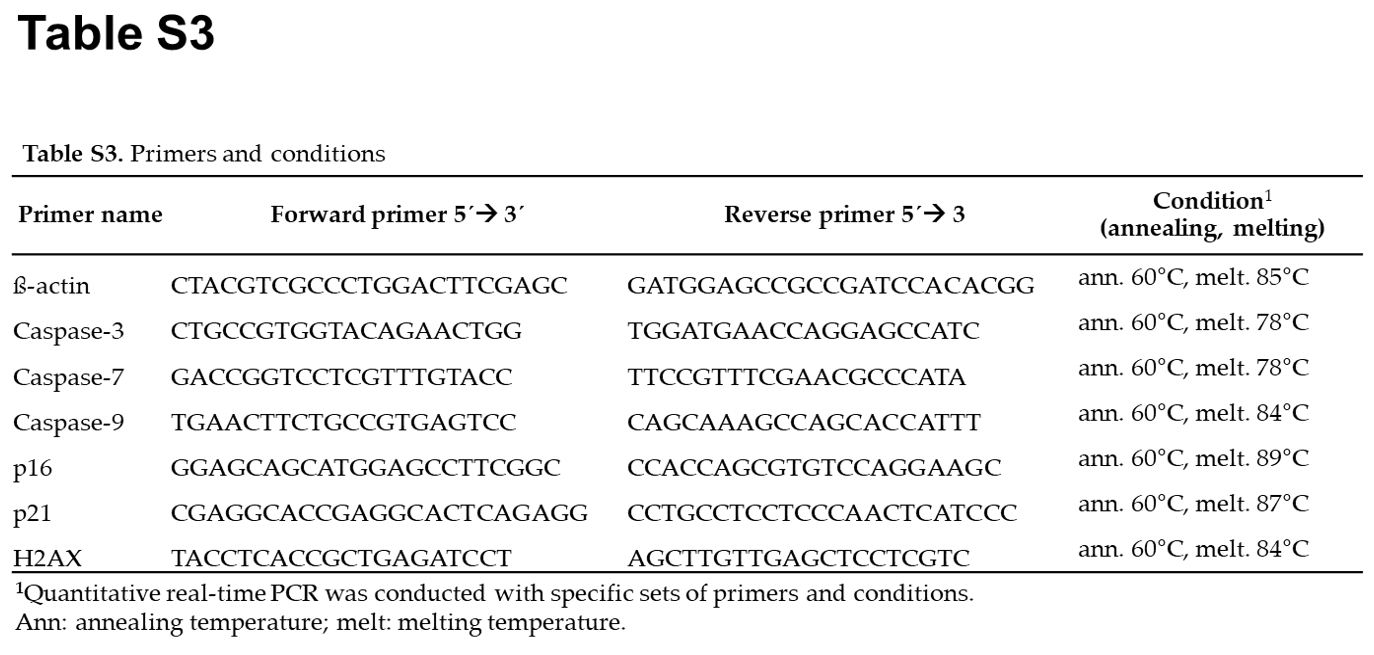

Supplement: Supplementary file 1 [file ijms-22-07886-s001.zip › Table_S3.tif]
